# Anti‐amyloid treatments: Why we think they are worth it

**DOI:** 10.1002/alz70861_108615

**Published:** 2025-12-23

**Authors:** Suzanne E. Schindler

**Affiliations:** ^1^ Washington University School of Medicine, St. Louis, MO USA

## Abstract

Years of experience watching our patients progressively decline and die from complications of Alzheimer's disease (AD) has strongly motivated us to provide newly approved anti‐amyloid treatments to appropriate patients. Following detailed and personalized discussions of the potential risks and benefits of these treatments with patients and their families, almost 300 patients at our clinic have chosen to receive lecanemab infusions. We have found the frequency and severity of complications, including amyloid‐related imaging abnormalities (ARIA), to be manageable and as expected based on clinical trials. While the longer‐term benefits of these treatments are not yet clear, our patients and their families are accepting of even a modest slowing of disease progression. We have experienced the complexities, burdens, costs, and major logistical challenges associated with the treatment of AD with anti‐amyloid treatments. However, we also understand that for some of our current patients with early symptomatic AD, anti‐amyloid treatments are their best option for fighting this devastating disease, and we find it worthwhile to provide these treatments to our patients.

